# Prevalence of Zoonotic Gastrointestinal Helminths Among Pet Dogs in Western Chitwan, Nepal

**DOI:** 10.1155/japr/9982123

**Published:** 2025-11-04

**Authors:** Sushil B. K., Saroj Adhikari, Shishir Regmi, Chet Raj Pathak

**Affiliations:** Faculty of Animal Science, Veterinary Science and Fisheries, Agriculture and Forestry University, Bharatpur, Nepal

**Keywords:** Bharatpur, helminths, Nepal, prevalence, zoonosis

## Abstract

Gastrointestinal helminth parasites (GIHPs) in dogs, belonging to the phyla Platyhelminthes and Nemathelminths, pose a zoonotic threat by contaminating human environments with cysts, oocysts, and eggs. Investigating GIHPs in domesticated dogs in close contact with humans is crucial to understanding zoonotic transmission. This study is aimed at assessing the prevalence of zoonotic helminths in dogs in western Chitwan, Nepal. A questionnaire survey was conducted among 240 canine caretakers. A total of 103 freshly defecated fecal samples were collected from three sites in Bharatpur Metropolitan City and preserved in 10% formalin. The samples were examined using sedimentation and flotation techniques. The study found an overall GIHP prevalence of 57.28% (59/103), identifying six parasite species. The most prevalent was *Ancylostoma* spp. (74.57%), followed by *Taenia* spp. (20.34%), *Toxocara* spp. (15.25%), *Strongyloides* spp. (5.08%), *Trichuris* spp. (3.39%), and *Dipylidium* spp. (3.39%). Multiple infections were observed in 15.25% (9/59) of cases. Infection rates in males and females were 61.02% and 38.98%, respectively. Among the puppies, adults, and older dogs, the infection rates were 60%, 57.97%, and 50%, respectively. By region, infection rates were 67.86% in Chanauli, 59.46% in Rampur, and 47.37% in Mangalpur. The dewormed (≤ 3 months) dogs had a 38.10% prevalence of gastrointestinal parasites (GIPs). In contrast, those dewormed less frequently (> 3 months) had a 56.10% prevalence, while nondewormed dogs had the highest prevalence at 68.29%. Out of 240 canine caretakers, only 50.41% were aware of parasitic zoonoses. The findings highlight the importance of regular deworming and veterinary intervention to control GIHPs and reduce the risk of zoonotic transmission, emphasizing the need for strategic health measures.

## 1. Introduction

Human beings are indeed living in an animal's world in the sense that lives are very much intertwined with a companion animal [1]. Among different companions, dogs around the world have always had very close relationships with humans [2]. Dogs are highly susceptible to infestation by gastrointestinal parasites (GIPs) because of their roaming nature in a free environment [3, 4]. Despite the close bonds between dogs and humans, dogs remain a major threat to public health as they are associated with more than 60 zoonotic diseases, including helminth parasites [4–6]. The exact number of GIPs transmissible between dogs and humans varies, but several notable zoonotic parasites exist [7]. Such GIPs are found in dogs worldwide but are more prevalent in developing countries where the communities are socioeconomically challenged [8]. Nowadays, people are sharing beds and utensils with their pet dogs, which is a potential risk of infection in humans [9]. The dog caretakers who are involved in feeding and handling the free roaming community dogs might be at risk. They may handle without proper precautions like wearing gloves, disinfectants, and handwashing after touching the dog and their fecal materials. One of the common GIPs acquired from the dog is hookworm (*Ancylostoma* spp.), which can penetrate intact human skin and migrate through subcutaneous tissues after contact with soil contaminated with infected animal feces [10]. The roundworm *Toxocara canis* can manifest as visceral larva migrans (VLM), ocular larvae migrans (OLM), and covert toxocariasis (CT) in humans [11]. Similarly, cestodes such as *Taenia* spp. and *Echioncoccus* spp. have dogs as definitive or reservoir hosts having tendencies to infect herbivorous domesticated, wild animals, and humans as well [12]. These intermediate stages may lead to diseased conditions like cysticercosis, hydatidosis, coenurosis, neurocysticercosis, and even deaths [13–17]. Nowadays, the number of pets is increasing globally, and the import and export of exotic breeds, and thus increasing the risk of zoonotic diseases [18]. Similarly, Bharatpur is also a rapidly developing metropolitan city located in the central-southern part of Nepal, and the number of pet owners is also increasing [19]. Previous research was accomplished in different locations in Nepal, but there is no information on zoonotic gastrointestinal helminth parasites (ZGIHPs) among dogs in Bharatpur [5]. Moreover, the knowledge of deworming and the potential public health issues related to ZGIHPs remain unaddressed.

To address the significant public health concerns due to gastrointestinal helminth parasites (GIHPs), this study is aimed at estimating the prevalence of these parasites in owned dogs in Chitwan. By shedding light on the current burden of GIHPs and bridging existing knowledge gaps, the findings could emphasize the need for regular deworming programs, improved veterinary care, and increased awareness to mitigate the associated public health risks.

## 2. Materials and Methods

### 2.1. Study Area

**Figure 1:**
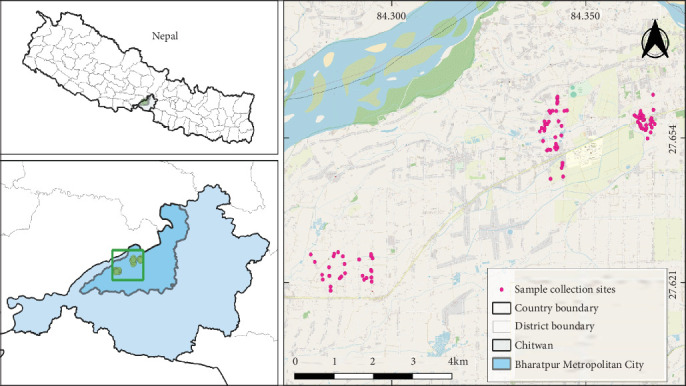
Different sampling spots for screening gastrointestinal parasites in dogs in the map of Nepal.

The cross-sectional study was carried out from November 2023 to February 2024. In Bharatpur metropolitan city of Nepal, located in the Chitwan district (latitude 27°36⁣′21.60⁣^″^ north, longitude 84°22⁣′47.28⁣^″^ east, and altitude of about 251 m above sea level (Figure 1). Bharatpur has a humid subtropical, dry winter climate, with an average temperature of 29.3°C (7.3% higher than Nepal's average). Annual precipitation is 252.9 mm (10 in.), with 182 rainy days (49.9% of the time) [20].

### 2.2. Sampling Technique

Animals recruited in the study were dogs owned by the owners, that is, pet dogs. The inclusion criteria included any dog regardless of age, sex, breed, etc. A questionnaire survey was conducted among 240 dog caretakers whose dogs were selected in the study regarding the awareness of zoonotic helminth parasites among pet dogs. Here, caretakers refer to the person who takes care of street or community dogs that might not have dogs of their own. Since it was not possible to collect fresh samples from the free-roaming community dogs of study sites, we excluded semidomestic dogs for sampling, and fecal samples were collected selectively from 103 intensively domesticated dogs.

### 2.3. Fecal Sample Collection From Dogs and Laboratory Examination

In total, 103 freshly voided fecal samples were collected in a ziplock poly bag containing 10% formalin for preservation. Each bag was well labeled, mentioning the breed of dog, age, sex, date, and time of collection. The samples were immediately transported in the cool box containing ice to the laboratory of the Department of Microbiology and Parasitology, Faculty of Animal Science, Veterinary Science and Fisheries (FAVF), Agriculture and Forestry University (AFU). Microscopic slides were prepared by the sedimentation and floatation technique [21]. Fecal samples were analyzed qualitatively by microscopic observation under magnifications of 100x and 400x. Each sample was analyzed in triplicate to minimize possible errors. The identification of the parasitic eggs and their measurements were performed using a micrometer scale [22]. The measurements have been performed with the help of digital camera software OPTICA PRO-VIEW Version x64 using an optical trinocular microscope (B-33LD1 optica Italy) equipped with a digital camera (OPTIKAM B3, Italy).

### 2.4. Data Management and Analysis

Statistical analysis was performed using the OpenEpi online source [23]. Pearson's chi-square test evaluated relationships between the result and specific explanatory factors. *p* < 0.05 was taken as statistically significant for all analyses. The data was visualized in the Google Colaboratory (Colab) environment [24].

## 3. Results

### 3.1. The Overall Prevalence of GIHPs in Dogs

The study indicated the overall prevalence of GIHPs in dogs to be 57.28% (59/103). Exclusively examining pet dogs, the infections were observed in 61.02% (36/59) male and 38.98% (23/59) female dogs. The sex difference was insignificant (*p* > 0.05) in GIP infection in dogs.

### 3.2. Identification of Different Species of GIPs in Dogs

**Figure 2:**
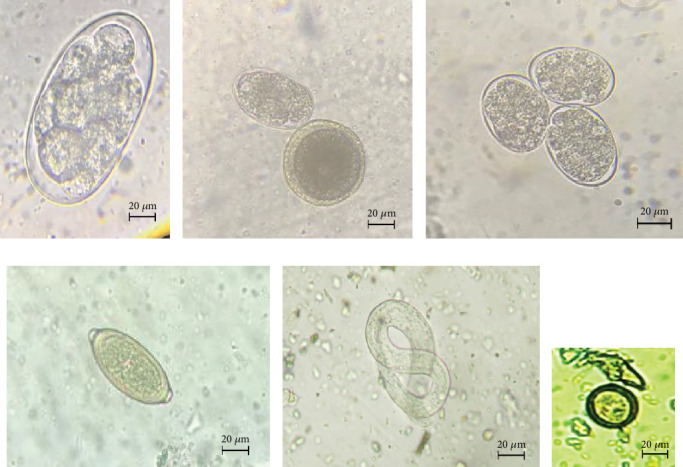
Parasitic eggs of different genera in the feces of dogs during microscopic examination at 400x magnification. (a) *Ancylostoma* sp., (b) *Ancylostoma* sp. upper and *Toxocara* sp. lower, (c) Strongyle type eggs, (d) *Trichuris* sp., (e) *Strongyloides* sp., and (f) *Taenia* sp.

The study revealed two classes (Nematoda and Cestoda) of six different genera of GIHPs (*Ancylostoma* spp., *Toxocara* spp., *Trichuris* spp., *Strongyloides* spp., *Dipylidium* spp., and *Taenia* spp.) (Figure 2, Table 1). *Ancylostoma* spp. was observed in 74.57% (44/59) of positive cases with the highest prevalence, whereas the lowest was 3.39% (2/59) for *Trichuris* and *Dipylidium.* About 98.30% (58/59) of positive infections were due to nematodes and 23.73% (14/59) were from cestodes. The above data adds more than 100% (72/59) due to multiple infections in some dogs. Multiple GI parasitic infections indicating two or three species in the same individual of 15.25% (9/59) were observed.

### 3.3. Age-Wise Prevalence of GIPs in Dogs

The age-wise prevalence showed that the higher infection of GIHPs in puppies of less than 1 year of age was 60% (12/20), followed by adult groups (1–6 years) at 57.97% (40/69), and least in old groups (> 6 years) at 50% (7/14) (*p* > 0.05).

### 3.4. Sex-Wise Prevalence of GIPs

The prevalence of GIPs was 56.25% (36/64) and 58.97% (23/39) in males and females, respectively. According to these results, the percentage of positive cases in females is slightly higher than in males, even if the positivity rates for the sexes are identical (*p* > 0.05).

### 3.5. Breed-Wise Prevalence of GIPs

**Figure 3:**
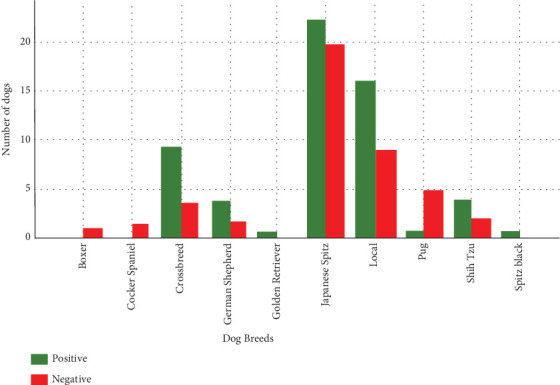
Breed-wise prevalence of gastrointestinal parasites in dogs.

The variations in the prevalence of GIPs across 10 dog breeds were observed (Figure 3). Crossbreeds had the highest prevalence at 75% (9/12), followed by local dogs at 64% (16/25). Japanese spitz showed a prevalence of 53.49% (23/43), while German shepherds had 66.67% (4/6), shih tzus had 66.67% (4/6), and pugs had a prevalence of 16.67% (1/6) each. Interestingly, boxers (0/1) and cocker spaniels (0/2) recorded no positive cases (0% prevalence). In contrast, golden retriever cross had 100% (1/1), and black spitz had 100% (1/1), with a prevalence of 100%; this was based on a single sample (*n* = 1) and therefore should be interpreted with caution.

### 3.6. Location-Wise Prevalence of GIPs in Dogs

Location-wise GIPs in dogs were prevalent at a descending rate in Chanauli 67.86% (19/28), Rampur 59.46% (18/37), and Mangalpur 47.37% (18/38) (*χ*^2^ = 2.87, *p* > 0.05, *df* = 2).

### 3.7. Deworming Practices and Prevalence of GIPs in Dogs

**Figure 4:**
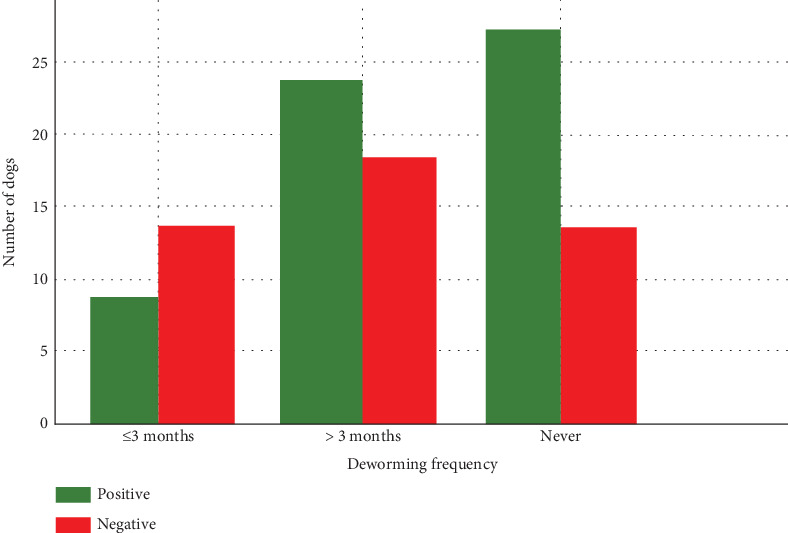
Prevalence of gastrointestinal parasites in dogs based on deworming history.

Out of 103 dogs studied, only 21 were dewormed within 3 months (regular interval), 41 had a duration of more than 3 months (irregularly), and the remaining 41 had no history of deworming (Figure 4). The prevalence of GIPs in dogs based on deworming history exhibited that 38.10% of dewormed dogs were infected by GIHPs, 56.10% of dogs that were irregularly dewormed were positive, and 68.29% of nondewormed dogs were positive (*χ*^2^ = 5.21, *p* = 0.073, *df* = 2).

### 3.8. Awareness of Dog Caretakers About Parasitic Zoonoses

Among the 240 dog caretakers, only 121 (50.41%) were aware of the parasitic zoonoses, especially due to helminth parasites, and the remaining were unknown.

## 4. Discussion

The risk of transmitting zoonotic helminth parasites has increased with the increasing number of pets and pet owners [4–6]. Close contact between pets, community dogs, and wild canines further elevates this risk [2]. Several factors contribute to the susceptibility of parasitic transmission, with the selection and administration of anthelmintic drugs for deworming pets playing a crucial role [7].

Studies on helminth parasites of dogs in many foreign countries are common; however, there are few documented research studies in Nepal. This is the first study to estimate the prevalence of GIPs in dogs in Bharatpur, Nepal. Previously, similar studies have been performed in other districts of Nepal: Kathmandu, Rupandehi, and Lalitpur [2, 25–27]. Several GIPs presented in this study pose zoonotic risks.

The parasite prevalence reported in our study (57.28%) is considerably higher than those observed in Kathmandu (46.7%) and lower than those observed in Lalitpur (95.7%) [27]. The results were comparable to earlier studies conducted in Rupandehi (58.75%) and Suryabinayak (59.50%), as well as in Italy (52.50%), Guimarães, Portugal (57.20%), and Mampong, Ghana (52.60%) [28]. These disparities in prevalence rates could be attributed to factors such as geography, diagnostic technique, sample size, sampling season, age, sex, deworming history, and awareness level.

The age-wise prevalence showed a higher infection of GIPs in puppies in comparison to adult and old dogs (*p* > 0.05), which is also supported by a previous study in Nepal [29]. This difference might be due to the underdeveloped immune system of younger puppies, prenatal, and lactogenic transmission [30, 31]. Females had a slightly higher infection rate (58.97%) than males (56.25%), but the difference was not statistically significant (*p* > 0.05). This finding is consistent with research conducted in Ethiopia [32]. During pregnancy, female reproductive hormones in circulation may trigger the dormant parasites in the somatic regions to be activated for further development [33].

In this study, seven species of intestinal helminth parasites were observed, which fall within the globally reported range of 5–10 species [25]. *Ancylostoma* was the most prevalent zoonotic parasite, which is supported by the findings of previous studies from Nepal, Mexico, and India [34]. The findings of this study are of high public concern because of the presence of various GIPs of zoonotic concern; the roundworm *Toxocara* and *Ancylostoma* could manifest as VLM and OLM [11]. Additionally, these dogs may serve as potential sources of infection for humans and other healthy dogs. The mixed GIP infections (15.25%) increase the risk of zoonotic transmission and make dogs more susceptible to further diseases.

The prevalence of GIPs did not vary significantly by location (*p* > 0.05). However, dogs in Chanauli had the highest infection rates compared to those in Rampur and Mangalpur. This may be due to a higher level of public awareness in Mangalpur compared to Chanauli regarding pet dog care, health management, and deworming practices.

The prevalence of GIPs in dogs based on deworming history showed the lowest in the dewormed dogs, followed by irregularly dewormed, and the nondewormed dogs (*p* > 0.05), which is supported by the previous studies of Nepal [25]. About half of the dog caretakers were aware of the gastrointestinal helminth parasitic zoonoses reflecting the need for public awareness in Nepal, which is supported by the previous report of Nepal [35]. This highlights the importance of deworming in reducing the risk of parasitic transmission in the Nepalese context. The high prevalence of parasites may be due to a lack of public awareness, careless dog ownership, and semidomestication practices. The primary contributing factor is the open sanitary system in the area. Additionally, poor hygiene, overcrowding, limited veterinary expertise, and lack of knowledge about zoonotic risks further increase the chances of disease transmission [12].

## 5. Conclusion

In conclusion, dogs in Bharatpur harbor a high prevalence of zoonotic parasites (57.28%) to which the residents are constantly exposed. The presence of mixed infection in the single host may imply an elevated environmental contamination with eggs of different parasites inside the house and other public places, creating a high risk of infection for humans, especially in children. Thus, the local authorities should implement the concept of one health, manage the proper sanitary system, and provide proper awareness to the pet owners and veterinary services within different wards of their metropolitan city to minimize the risk to public health.

## Figures and Tables

**Table 1:** Species of GIHPs found in western Chitwan.

**Parasites classes**	**Parasitic helminth species**	**Positive (number of dogs)**	**Positive (%)**
Nematoda	*Ancylostoma* spp.	44	74.57
	*Strongyloides* spp	3	5.08
	*Toxocara* spp.	9	15.25
	*Trichuris* spp.	2	3.39

Cestoda	*Dipyllidium* spp.	2	3.39
	*Taenia* spp.	12	20.34

## Data Availability

The original data for this study are available from the corresponding author.
